# Is Motor Learning Mediated by tDCS Intensity?

**DOI:** 10.1371/journal.pone.0067344

**Published:** 2013-06-24

**Authors:** Koen Cuypers, Daphnie J. F. Leenus, Femke E. van den Berg, Michael A. Nitsche, Herbert Thijs, Nicole Wenderoth, Raf L. J. Meesen

**Affiliations:** 1 BIOMED - Biomedical Research Institute, Hasselt University, Diepenbeek, Belgium; 2 REVAL - Research Institute, PHL University College, Diepenbeek, Belgium; 3 Motor Control Laboratory, Department of Biomedical Kinesiology, Katholieke Universiteit Leuven, Heverlee, Belgium; 4 Department of Clinical Neurophysiology, Georg-August University, Göttingen, Germany; 5 I-BioStat - Interuniversity Institute for Biostatistics and statistical Bioinfirmatics, Hasselt University, Diepenbeek, Belgium; 6 Katholieke Universiteit Leuven, Leuven, Belgium; 7 Neural Control of Movement Lab, Department of Health Sciences and Technology, Eidgenössische Technische Hochschule Zürich, Zürich, Switzerland; Hospital Nacional de Parapléjicos, Spain

## Abstract

Although tDCS has been shown to improve motor learning, previous studies reported rather small effects. Since physiological effects of tDCS depend on intensity, the present study evaluated this parameter in order to enhance the effect of tDCS on skill acquisition. The effect of different stimulation intensities of anodal tDCS (atDCS) was investigated in a double blind, sham controlled crossover design. In each condition, thirteen healthy subjects were instructed to perform a unimanual motor (sequence) learning task. Our results showed (1) a significant increase in the slope of the learning curve and (2) a significant improvement in motor performance at retention for 1.5 mA atDCS as compared to sham tDCS. No significant differences were reported between 1 mA atDCS and sham tDCS; and between 1.5 mA atDCS and 1 mA atDCS.

## Introduction

Recently, transcranial direct current stimulation (tDCS) has been shown to be effective for improving motor learning [1], [2] and enhancing motor recovery in healthy subjects and patients suffering from neurological diseases such as stroke [3]–[5] and Parkinson’s disease [6].

Electrophysiological data suggest that direct current stimulation elicits polarity-dependent and long-lasting cortical excitability changes outlasting the stimulation period by up to 90 min [7], [8]. Furthermore, tDCS is presumed to strengthen synaptic connections through a mechanism similar to long-term potentiation (LTP), a cellular mechanism that underlies learning [9], [10]. Fritsch et al. (2010) proposed that tDCS might improve motor skill learning through augmentation of synaptic plasticity within the primary motor cortex (M1) [11]. Previous work demonstrated that M1 participates in both fast on-line learning [12], [13] and in early stages of consolidation in motor sequence learning [14].

Whereas several studies reported clinically meaningful beneficial effects of a single session of 1 mA anodal tDCS (atDCS) in patient populations[3]–[6], less strong effects are reported in studies conducted in healthy subjects. Until now, a current intensity of 1 mA for anodal tDCS (atDCS) was applied during motor learning experiments. Optimizing strategies to enhance the efficacy of tDCS are needed. Previous electrophysiological [7] and cognitive studies in patients [15] and healthy humans [16], [17] suggest that increasing stimulation intensity might be a valuable approach, since the efficacy of stimulation seems to depend on intensity. Therefore, the present study aims to reveal the effects of increasing stimulation intensity on motor learning in healthy subjects under the hypothesis that higher stimulation intensity leads to enhanced skill acquisition.

## Materials and Methods

### Ethics Statement

Subjects provided written informed consent and experimental procedures were approved by the Central Ethics Committee of UZ Leuven and the local Ethics Committee of the University of Hasselt. The study conforms to the principles stated in the Declaration of Helsinki.

Thirteen healthy subjects (mean age of 19.92±1.12 years; 7 males) participated in this double-blinded crossover study. Eleven subjects were right-handed (mean lateralization quotient: 79.58±20.84) and 2 were left-handed (mean lateralization quotient: −80.00±28.28) according to the Edinburgh Handedness inventory [18].

**Figure 1:**
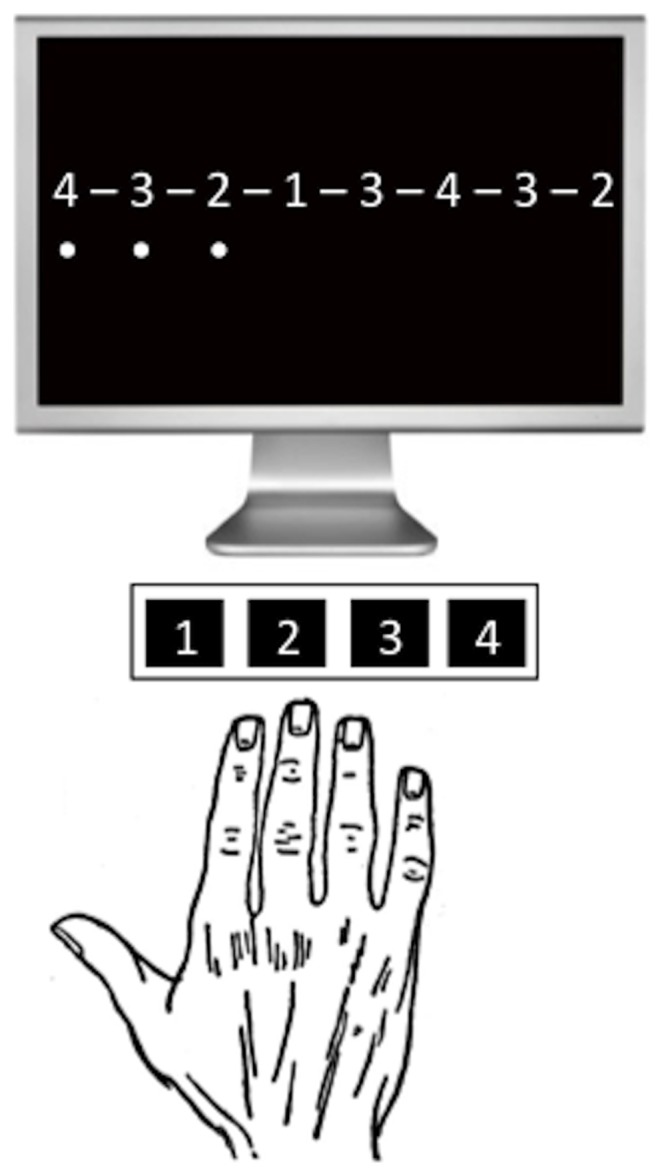
Subjects were instructed to perform a 8-element finger sequence with the dominant hand by pressing different keys, each corresponding to one of the four fingers (2nd–5th).

In three pseudo-randomized, counterbalanced sessions separated by at least 3 days, subjects received either atDCS (HDCstim, Newronika, Italy) with an intensity of 1.5 mA, 1 mA or sham tDCS for 20 min on the primary motor cortex (M1) contralateral to the dominant hand while performing a unimanual motor learning task. In the sham condition, the electrode montage was identical to the real stimulation conditions and electrodes were also attached for 20 min, however subjects only received current during the first 26 sec. More specifically, the current was ramped-up for 7 sec, followed by 12 sec of 1 mA atDCS and then ramped-down for 7 sec. The anode (surface: 25 cm^2^, current density of 0.04 mA/cm^2^ for 1 mA atDCS and 0.06 mA/cm^2^ for 1.5 mA atDCS) was centered at the hotspot of the first dorsal interosseous muscle, as determined by transcranial magnetic stimulation. The cathode size (surface: 50 cm^2^, current density of 0.02 mA/cm^2^ for 1 mA atDCS and 0.03 mA/cm^2^ for 1.5 mA atDCS) was increased to make the electrode functionally inert [19] and was fixed over the supraorbital region of the other hemisphere. Subjects were instructed to perform a finger sequence task with the dominant hand by pressing different keys (see, Fig. 1), each corresponding to one of the four fingers (2nd–5th). The sequences were [4 2 1 3 4 2 3 2], [2 4 2 1 3 2 3 4] and [2 4 3 1 2 3 2 4] (1 = index, 2 = middle, 3 = ring and 4 = little finger). The practiced sequence was displayed on the screen and a black dot appeared on the screen whenever a key was pressed. No feedback about the correctness of the performance was provided. Button presses were recorded using E-Prime (E-prime v2.0, Psychology Software Tools Inc., PA, USA]. In a single block sequences were initiated within a 30 second time frame, followed by a 30 second resting period. Each block was terminated after completion of the last sequence.

Subjects were instructed to perform as many sequences as possible. The amount of sequences provided during each block depended on the speed of the subject. In other words, when a subject performed faster in a block, a larger amount of sequences was provided within that block. Each session consisted of 26 blocks. 3 blocks (baseline, 3 min) were provided before application of the stimulation, followed by 20 training blocks (20 min) under atDCS/sham tDCS; and finally 3 blocks (post-intervention, 3 min) were administered 30 min after the stimulation. All sequences initiated during the 30 sec practice block were considered for analysis and a motor performance score was calculated by dividing the percentage of correct sequences by the mean inter tap interval ( = the average time between two successive key presses), thus considering both the speed and accuracy requirements. After each session, the level of attention, fatigue and perceived discomfort during the session was rated by the visual analogue scale (VAS). Furthermore subjects were asked to report the amount (hours) of previous night’s sleep and sleeping quality (VAS).

Advanced linear models applications (SAS 9.2, SAS institute Inc., Cary, NC) were used for statistical analysis. Prior to analysis, the motor performance score was normalized (%) to baseline for each subject separately. To analyze performance differences between conditions at a single (time) point, a paired t-test was applied. The bonferroni correction was used to correct for multiple comparisons. To evaluate the effect of stimulation intensity during motor learning over time, a mixed model including fixed effects for INTENSITY (1 mA atDCS, 1.5 mA atDCS and sham), and TIME (20 training blocks) and their interaction was used to estimate the rate of change (i.e. slope-analysis) of motor performance. Statistical power and sample size calculations were carried out for the evolution of the slope and at post-intervention. The significance level was set at p<.05.

## Results

At baseline, paired t-tests revealed no significant differences in motor performance between the different stimulation conditions (all, p>0.05).

**Figure 2:**
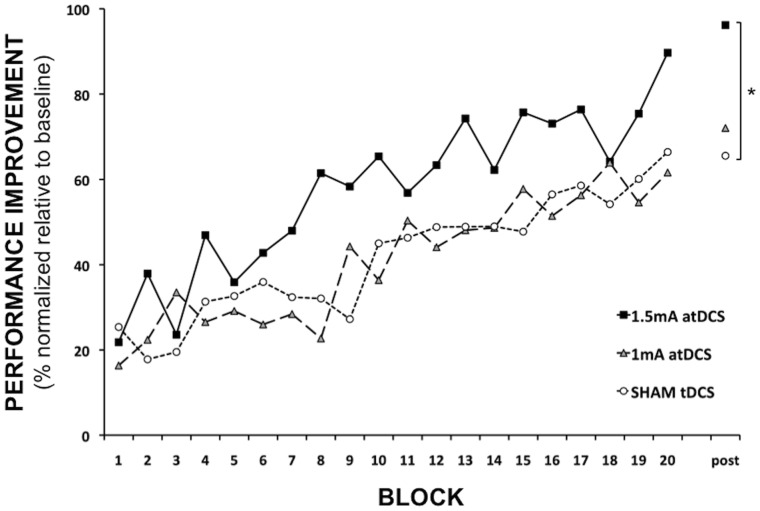
Evolution of motor performance during motor learning and at post-intervention (% normalized relative to baseline) for the 1.5 mA atDCS, 1 mA atDCS and sham tDCS condition.

During motor learning (20 blocks), a significant INTENSITY × TIME interaction was reported (F = 4.32, p = 0.014). This result indicates that the slopes are significantly different (see, Fig. 2) for the different tDCS intensities. The slope was significantly steeper for 1.5 mA atDCS as compared to sham condition [Difference in slope estimates for 1.5 mA atDCS as compared to sham: 7.22 (StDev. = 2.49), p = 0.004], indicating that motor learning occurred faster during 1.5 mA atDCS. No significant difference in slope was reported between 1.5 mA and 1 mA [Difference in slope estimates for 1.5 mA atDCS as compared to 1 mA atDCS: 3.93 (StDev. = 2.34), p = 0.092]; and between 1 mA atDCS and sham [Difference in slope estimates for 1 mA atDCS as compared to sham: 3.29 (StDev. = 2.54), p = 0.20).

At post-intervention (see, Fig. 2), a paired t-test revealed a significant difference in motor performance for 1.5 mA atDCS as compared to sham (p = .044). No significant difference was found between 1.5 mA and 1 mA atDCS (p = 0.08) and between 1 mA atDCS and sham (p = 0.34).

The results mentioned above should be interpreted in combination with the power and sample size calculations as shown in Table 1.

**Table 1:** For all contrasts the values for the effect size, power and the required sample size to reach a power of 0.80 are reported.

	Slope			Post-intervention		
	Effect Size	Power	Sample size (Power = 0.80)	Effect Size	Power	Sample size (Power = 0.80)
1.5 mA atDCS vs. 1 mA atDCS	28	0.29	63	24	0.40	39
1.5 mA atDCS vs. SHAM tDCS	23	0.43	35	30	0.67	18
1 mA aTDCS vs. SHAM tDCS	4.5	0.10	593	6.5	0.11	445

Results for the evolution of the slopes and at post-intervention are shown. Note that the effect size is defined as the absolute value of the mean difference between two groups.

No significant differences in the amount of previous night’s sleep, sleep quality, level of attention, level of fatigue and level of discomfort were reported between conditions (all, p>0.05; see Table 2).

**Table 2:** Mean (StDev) sleep (duration and quality) and level (0 = low, 10 = high) of attention, fatigue, and discomfort perceived during each session (SHAM tDCS, 1 mA atDCS and 1.5 mA atDCS).

	SHAM tDCS	1 mA atDCS	1.5 mA atDCS
Sleep (hours)	7.46 (1.42)	7.96 (1.03)	7.23 (1.13)
Sleep (quality)	6.85 (2.58)	7.08 (1.55)	8.00 (1.35)
Attention	7.08 (1.26)	7.08 (1.60)	7.62 (0.65)
Fatigue	3.31 (2.66)	2.92 (2.79)	2.69 (2.36)
Discomfort	1.61 (1.56)	2.15 (2.38)	0.92 (0.86)

No significant differences were reported between sessions (all, p>0.05).

## Discussion

The present study reveals that a combination of motor learning and 1.5 mA atDCS over M1 contralateral to the (dominant) hand performing the motor task leads to a significant improvement of motor performance as compared to sham stimulation in healthy subjects. Remarkably, this effect was seen both during motor training and at post-intervention (30 min after stimulation). Although the effects of a single session of atDCS on motor learning in healthy individuals have been studied previously [20], [21], this is the first study evaluating the stimulation intensity-dependent effects of atDCS intensity on motor learning.

Our results are in line with Boggio et al. (2006) who reported a significant improvement in working memory performance in Parkinson’s disease patients when applying 2 mA atDCS on the left dorsolateral prefrontal cortex, whereas 1 mA atDCS or sham stimulation did not result in significant effects [15]. In contrast, Nitsche et al. (2003) showed a significant shortening of absolute reaction time during a serial reaction time task (SRTT) after a single session of 15 min of 1 mA atDCS over M1 as compared to sham stimulation in healthy subjects [21]. Similar results were also reported by a recent study of Kantak et al. (2012) reporting decreased reaction time in a SRRT during (online) and after (offline) atDCS in healthy adults. In contrast, our results showed no performance differences between 1 mA atDCS and sham after motor learning [22]. Whereas Nitsche et al. (2003) and Kantak et al. (2012) used a protocol evaluating reaction time [21], [22], the current results are obtained using a compound measurement assessing performance as function of both accuracy and speed. Since both parameters influence each other, the current protocol does not allow disentangling accuracy and speed and therefore we cannot attribute performance to these parameters independently. The absence of a performance difference between 1 mA atDCS and sham in the present study is in line with Boggio et al. (2006), who showed no significant effect of sham or 1 mA atDCS over the dominant M1 on fine motor skill performance in healthy subjects [20]. Although we expected to find a significant improvement of motor performance during 1.5 mA atDCS as compared to the 1 mA atDCS condition, only a non-significant trend was reported. This finding is probably due to the relative small sample size.

In the current study we did not evaluate the physiological changes underlying changes in motor performance. Previous findings provide evidence that increased stimulation intensity will lead to increased excitability of the area (M1) under the anode during and after atDCS [23]. Furthermore, Nitsche & Paulus (2000) reported that the size and endurance of excitability changes after atDCS depended on stimulation duration and current intensity [7]. More specifically, increasing either intensity or duration led to prolonged and larger after effects. Therefore it might be speculated that larger current intensity leads also to increased strengthening of learning-related synaptic connections, thus resulting in improved performance. On the contrary, Antal et al. (2007) reported decreased excitability after atDCS when atDCS was associated with motor excercise, showing that tDCS-induced plasticity is highly dependent on the state of the subject during stimulation [24]. Future studies are needed to clarify these findings.

In conclusion, this study demonstrates (1) a significant improvement in online and (2) offline performance for 1.5 mA atDCS as compared to sham tDCS. No significant effects were reported between 1 mA atDCS and sham tDCS; and between 1.5 mA atDCS and 1 mA atDCS. Although only a trend was reported between 1.5 mA atDCS and 1 mA atDCS, our results indirectly support the hypothesis that stimulation intensity plays an important role in obtaining the desired result. Increasing the sample size and/or current intensity (for example 2 mA or more) might lead to increased effects between conditions.
